# Self-Management of Oxaliplatin-Related Peripheral Neuropathy in Colorectal Cancer Survivors

**DOI:** 10.1155/2013/547932

**Published:** 2013-08-25

**Authors:** Cindy Tofthagen, Laura Gonzalez, Constance Visovsky, Alex Akers

**Affiliations:** ^1^University of South Florida College of Nursing in Tampa, FL 33612, USA; ^2^University of Massachusetts Boston, Boston, MA 02125, USA; ^3^Dana Farber Cancer Institute, Boston, MA 02215, USA; ^4^University of Central Florida, Orlando, FL 32816, USA

## Abstract

*Purpose*. The purpose of this study was to evaluate medications that cancer survivors with oxaliplatin-induced peripheral neuropathy take to control neuropathic symptom, and to explore self-management techniques used at home to provide temporary relief of painful neuropathy. This was a mixed methods, descriptive, cross-sectional study using self-reported data from colorectal cancer survivors previously treated with oxaliplatin. We analyzed demographic and medication data obtained from participants, along with written comments from an open-ended question regarding methods participants had tried to self-manage symptoms of neuropathy. *Results*. Twenty-nine percent of the sample reported taking some type of nutritional supplement with potential neuroprotective qualities. Opioids were being taken by 10% of the sample, and nonsteroidal anti-inflammatory and over-the-counter medications were taken by 15% of participants. Twelve percent of participants were taking antidepressants and 10% were taking anticonvulsants, primarily gabapentin. Recurrent themes for nonpharmacologic treatment included avoiding the cold/keeping warm, keeping moving, massaging or rubbing the affected area, and living with it. *Conclusions*. Patients treated with oxaliplatin for colorectal cancer utilize a variety of traditional pharmacologic agents and nutritional supplements in an effort to self-manage neuropathic symptoms. Patients also employ a variety of home-based therapies to provide temporary relief of peripheral neuropathy symptoms.

## 1. Introduction

Oxaliplatin is a highly neurotoxic chemotherapy drug routinely used to treat colorectal cancer in the adjuvant and metastatic disease settings [1]. Among the approximately 100,000 persons diagnosed with colon cancer each year, approximately 60% will be candidates to receive oxaliplatin-based chemotherapy [2]. At least 48% of patients who receive oxaliplatin will develop chronic peripheral neuropathy that arises during treatment, and still more patients will go on to develop chronic neuropathy after cessation of treatment, a phenomenon known as “coasting” [3–6].

Few evidence-based interventions exist for treatment of oxaliplatin-related neuropathy [7–9]. Supportive care for peripheral neuropathy includes pain management, fall prevention, home safety education, provision of psychological support, measures to enhance or maintain physical functioning, and recommendations for assistive devices to help with daily activities [10]. In clinical practice, the focus of management of peripheral neuropathy is primarily aimed at control of neuropathic pain. Agents such as anticonvulsants, antidepressants, and opioids are commonly prescribed for treatment of neuropathic pain in individuals with peripheral neuropathy. However, untoward side effects such as somnolence and weight gain may result in poor adherence [11, 12]. A lack of understanding concerning the treatment regimen may result in patients taking anticonvulsants and antidepressants on an as-needed basis, instead of around the clock, as normally prescribed for neuropathic pain, resulting in ineffective pain control. Frequent titration of medications prescribed for neuropathic pain is required to achieve adequate pain control but patient-provider communication often breaks down before optimal dosing is achieved [13–15].

Although calcium and magnesium infusions may help prevent development of oxaliplatin-related peripheral neuropathy [16–19], data supporting use of other nutritional supplements for either prevention or treatment of oxaliplatin-induced peripheral neuropathy is sparse [20–22]. However, well-meaning clinicians, in the absence of other tolerable and effective treatments, may recommend a variety of nutritional supplements, including vitamin E, B vitamins, alpha lipoic acid, acetyl-L-carnitine, and oral calcium and magnesium to patients seeking relief from symptoms of peripheral neuropathy. Patients also may self-prescribe nutritional supplements because they are readily available and perceived to be harmless or relatively free of side effects.

Patients may also try a variety of nonpharmacologic methods at home to relieve their neuropathy symptoms, with varying degrees of success. Previous work in persons with acquired immune deficiency syndrome (AIDS) related neuropathy suggests that taking hot baths, resting from activity, applying foot cream, elevating feet, and exercising may provide temporary relief of painful neuropathy [23, 24]. Similar research about self-management strategies has been conducted in breast cancer patients receiving taxane chemotherapy. Results demonstrate that patients developed varied, individual coping strategies for each impacted activity such as driving, walking, socializing, cooking, balance, sleeping, and work. These interventions included manipulating their movements (walking slowly), making changes within their environment to prevent exacerbation of symptoms (taking baths instead of showers), and maintaining a positive attitude [25].

Our research team previously described persistent problems with peripheral neuropathy and associations between peripheral neuropathy, health-related quality of life, sleep disturbance, and depressive symptoms in colorectal cancer survivors as far as 7 years after treatment [26]. The purpose of this study was to evaluate medications that cancer survivors with oxaliplatin-induced peripheral neuropathy take to control neuropathic symptoms and to explore self-management techniques that participants used at home to provide temporary relief of painful neuropathy.

## 2. Methods

### 2.1. Study Design

This was a descriptive, cross-sectional study using self-reported data from colorectal cancer survivors previously treated with oxaliplatin-based chemotherapy. To conduct this analysis, we used demographic and medication data obtained from participants, along with written comments from an open-ended question regarding methods participants had tried to self-manage symptoms of neuropathy.

### 2.2. Sample and Setting

Men and women with a history of stage III-IV colorectal cancer treated with oxaliplatin at the Moffitt Cancer Center between 1 and 8 years before enrollment were included in this study. Diabetics and individuals with preexisting neuropathy (before receiving chemotherapy) were excluded from the study. To be eligible for the study, participants met the following eligibility criteria:

1. at least 18 years of age; treated with oxaliplatin for stage III-IV colorectal cancer;
2. no documented or psychiatric or neurologic disorders that would interfere with study participation;
3. able to speak and read standard English;
4. provide informed consent.

### 2.3. Procedures

The study was approved by the Institutional Review Board at the University of South Florida and patients were identified through the Moffitt Cancer Center Cancer Registry. Survivors meeting the study criteria were contacted by telephone to confirm eligibility and to obtain informed verbal consent. Enrolled participants were mailed a study packet and a postage paid return envelope. Approximately 1 week later, those survivors who had not yet returned the packet were contacted by telephone and prompted to complete and return the material. A total of 128 survivors agreed to participate. Of these, 111 (94%) survivors completed and returned the study packet.

## 3. Measures

Demographic data were obtained via a standard self-report questionnaire. Demographic variables included gender, age, race/ethnicity, annual household income, educational level, and marital status. Participants were asked to provide a list of all current medications and to provide an open-ended response to the following question: “If you have numbness, tingling, or discomfort in your hands or feet, muscle weakness, joint or muscle pain, or loss of balance, what have you tried to relieve your symptoms? How helpful was it?” Questionnaires that were also completed by participants and reported on in an earlier publication included a modified version of the Chemotherapy-Induced Peripheral Neuropathy Assessment Tool [27], the Center for Epidemiological Studies-Depression Scale (CES-D) [28], the Insomnia Severity Index (ISI) [29], and the Medical Outcomes Study Short Form-36 [30]. Medical data were reviewed at the completion of study participation to obtain information about the number of cycles and cumulative doses of oxaliplatin; however, cycle number and dosing information were not available for the majority of participants.

### 3.1. Data Analysis

Qualitative descriptive methodology, with the written narrative responses as the method of inquiry, was used to explore the medications and home-based strategies used by colorectal cancer survivors in the self-management of oxaliplatin-related peripheral neuropathy. This analytic strategy was chosen since little is known about self-management of oxaliplatin-related peripheral neuropathy, and thus, descriptive information is needed [31].

Qualitative data were analyzed using Atlas Ti [32], a qualitative data management software package used for categorization, thematic coding, and assessment of meanings and relationships within text. Transcripts were proof-read, reviewed for accuracy, and coded and sorted into categories reflecting significant elements of the responses. Categories were coded into themes and subthemes [33]. Codes and categories were checked for accuracy by the researcher and colleagues (oncology researcher and qualitative researcher) to ensure accuracy and to increase validity.

Medications were examined to identify those commonly used for treatment of neuropathic pain (anticonvulsants, antidepressants, opioids, and nonsteroidal anti-inflammatories) or are purported to offer some degree of neuroprotection (nutritional supplements). These medications were categorized and descriptive statistics including frequencies and percentages were analyzed using Microsoft Excel software. Two researchers conducted independent analyses of the medication data to confirm accuracy of results.

## 4. Results

A total of 128 survivors agreed to participate. 111 (94%) survivors completed and returned the study packet and 105 (82%) provided a list of current medications including over-the-counter medications and nutritional supplements. Seventy percent (*n* = 78) provided written comments regarding what they have tried for neuropathy (70%). The sample was 51% male, 88% Caucasian, 71% stage 3-colon cancer, and 37% still employed (not retired or disabled) [26]. Time since beginning oxaliplatin-based chemotherapy ranged from 1 to 7 years with a mean of 3 years since oxaliplatin-based chemotherapy was initiated.

Twenty-nine percent (*n* = 30) of the sample reported taking some type of nutritional supplement with potential neuroprotective qualities. B-vitamins, alpha lipoic acid, acetyl-L-carnitine, L-glutamine, Bing Ling Herbals, omega-3 fatty acids, oral calcium, and magnesium were among the nutritional supplements used. Opioids were being taken by 10% (*n* = 11), and nonsteroidal anti-inflammatory and over-the-counter medications such as acetaminophen or ibuprofen were taken by 15% (*n* = 16) of participants. Twelve percent of participants (*n* = 13) were taking antidepressants and 10% (*n* = 10) were taking anticonvulsants, primarily gabapentin. Recurrent themes for nonpharmacologic treatment included avoiding the cold/keeping warm, keeping moving, massaging or rubbing the affected area, and living with it.  Table 1 contains quotes pertinent to recurrent identified themes. In addition to the themes identified and data of medication use, participants made reference to the use of general health maintenance strategies such as “eat healthy, light exercise, and keep emotionally calm.” Participants also mentioned the use of acupuncture and anodyne light therapy as self-management strategies they had tried at home to help alleviate symptoms.

## 5. Discussion

This study examines specific medication and self-care strategies for the treatment of oxaliplatin-based peripheral neuropathy in colorectal cancer survivors. Participants in this study employed a variety of complementary and alternative approaches to control neuropathic symptoms, which are similar to the self-care strategies reported by patients of earlier studies with AIDS-related neuropathy and those receiving taxanes [23, 24].

### 5.1. Medications

Relatively few participants were taking anticonvulsants or antidepressants, which are considered first-line treatment for neuropathic pain [13–15]. Similarly, Gharibian et al. (2013) [11] found that patients taking antidepressants or anticonvulsants for neuropathic pain had less than a 45% compliance rate and that less than 25% consistently refilled their prescriptions for these drugs. Reasons patients may choose not to take these classes of medications for treatment of neuropathic pain may include low tolerability, perceived risks associated with these medications, lack of perceived benefit, misunderstanding of how antidepressants or anticonvulsants can be helpful, or confusion regarding dosing and need for dose escalation. A limitation of this study is that its cross-sectional design does not allow for examining patterns in use of medications over time or provide insight as to how many participants had been prescribed medications but elected either not to take them at all or to discontinue them.

Nutritional supplements were more frequently taken by participants than any other type of medication we examined, even though there is limited empirical evidence to support the use of any specific nutritional supplements for treatment of existing neuropathy in cancer survivors. We do not know whether these were recommended by a healthcare provider or whether patients independently sought out information on the use of nutritional supplements. Patients may seek alternative forms of treatment when they perceive that the risks and side effects associated with standard medical treatments are too high or that available treatments are of limited benefit, although we did not specifically collect data regarding the reason they were taking.

### 5.2. Self-Care Strategies

Participants reported that cold temperatures exacerbated painful neuropathies, caused stiffness, and further impaired physical functioning. Self-management strategies for keeping the affected areas warm were often utilized. Wearing gloves and socks, running hot water over the affected areas, use of heating pads, and drinking warm liquids were strategies that patients used to keep warm. Primarily, symptoms associated with cold temperatures, including dysphonia, laryngospasm, and electric shock sensation when touching a cold object, have been associated with acute oxaliplatin neurotoxicity [34]. Our data indicates that cold temperatures may also increase symptoms of neuropathy well past the acute treatment phase, and suggesting methods to keep the entire body warm as well as the affected extremities may help to minimize neuropathy symptoms on a temporary basis. However, the data from this study does not permit an analysis of how effectively keeping the affected areas warm may have mitigated neuropathic symptoms.

Cold-induced neuropathic symptoms may not follow classic distal to proximal distribution. One participant described symptoms in the hands but none in the feet; “*I have numbness in my fingertips only when they get cold. I have muscle weakness in my fingers (doing things like opening lids, containers, writing, etc.) when they are cold. The weakness is significant. I have a decrease in fine motor skills when my fingers are cold. At normal temperatures, I have no symptoms. It is a definite change in how they were prior to the chemo. My feet are fine I have no remaining symptoms.*” Some patients obtained temporary relief by massaging or rubbing the affected area of the body and others reported a feeling that there was little in the way of medical or self-management that provided substantial benefit, concluding that neuropathy is something that they must learn to cope with and endure.

Participants reported using various types of physical activity and exercise to help alleviate peripheral neuropathy symptoms to permit them to function at prechemotherapy levels. Participants described utilizing general physical activities and exercise such as yoga, swimming, biking, jogging or running, lifting weights, walking, golfing, physical therapy, a period. Some participants provided more specific descriptions of their exercise regimens including “*Maintained prechemotherapy fitness program by jogging 1.5–2 miles 3 times a week*” and “*for balance, practice standing on one leg and alternating legs*.*”*

Exercise can be safely recommended to patients with neuropathy, specifically those suffering from impaired balance [35], but as with other cancer-related symptoms, patients may benefit from having specific recommendations for type of exercise, dose, and frequency. Recommendations for referral to physical therapy may be warranted as well as beneficial in assisting patients to maintain or regain functional performance levels after treatment with oxaliplatin. While there is a potential role for physical activity and exercise in the management of peripheral neuropathy, the types of activities and exercise that may be most beneficial, frequency, and duration are not known. Further research of physical activity and exercise is critically needed, as pharmacologic strategies for managing peripheral neuropathy have not proven universally successful.

Although strategies like massage, pain medication, and application of heat may provide temporary symptom relief, participants also expressed the feeling that there was little they could do to relieve their symptoms, a view that may have been supported by healthcare providers in some cases. One participant wrote “*Doctor said there is no cure or relief*.” Another participant expressed gratitude for ongoing research in the area with the following statement included with the questionnaire packet, “*Thank you so much for pursuing this research. I have asked to speak with Sanofi research and development to advise them of the profound side effects post oxaliplatin. I have had no response from them. Thank you for this research, my internet research only gives outcome date up to 2 years after treatment. I sense there are many clients that have profound chemo deficits long after 2 years.*”

## 6. Conclusions

Patients treated with oxaliplatin for colorectal cancer utilize traditional pharmacologic agents and nutritional supplements in an effort to self-manage neuropathic symptoms. Patients also employ a variety of home-based therapies to provide temporary relief of peripheral neuropathy symptoms, with minimum efficacy. It is clear that patients want and need strategies to assist them with self-management of neuropathic consequences of cancer treatment. Future research testing the various means of self-management, including massaging, keeping the affected area warm, and generating “whole body” warmth and comfort, is indicated. Additionally, the development and testing of a sequential approach to the self-management of oxaliplatin-related neuropathy may be helpful in identifying which strategies are appropriate sooner after treatment as opposed to those that may be more effective when “coasting” of the chemotherapeutic agent has taken place. Additional research is needed to identify new methods of managing symptoms of oxaliplatin-related neuropathy at home that will provide effective symptom relief as well as be acceptable and tolerable to patients.

## Figures and Tables

**Table 1:** Treatments patients have tried to relieve symptoms of neuropathy.

Medication-related comments	Neurontin—did not help at all
	I'm taking Neurontin but I'm not sure it really helps
	Tried L-carnitine and B-complex for about two weeks and one didn't agree with my stomach as many pills dont, so I just decided i didn't need any more problems. All joints ache and take Advil liquid once in a great while.
	Have numbness tingling ant discomfort in hands and feet. Tried XS Tylenol.
	Vitamin B6 not helpful
	Gabapentin I often wonder that it really helps?? Nerve heals it self??
	Gabapentin at 2700 mg daily relieves the tingling in my hands and helps relieve the tingling in my feet. Does not improve numbness. If I forget a dose I get stabbing pains in my fingers. When Istopped Vitamin B1 symptoms seemed to get worse.
	I've tried Lyrica and amitriptyline in treating upward doses which both messed with my head so I quit. What relieves the pressure, tingling, pain the best is a combination of gabapentin and Oxycontin, which last up to 5 hours. I have recently decreased the dose of Oxycontin to 10 mg 3× daily and hopefully I can quit. But some days the pressure becomes too great. I wish this would go away, been to long.
	Calcium provided marked difference immediately, Potassium slight difference, Tylenol short-term relief, Neurontin 300 mg 3× daily not helpful, Hydrocodone managed pain, Multivitamins marked difference immediately.
	I talked wit my chemo oncologist about relieving the pain from the neuropathy. My doctor prescribed me Neurontin but warned if I didn't see a reduction in symptoms it likely wouldn't help. I took Neurontin for 3 weeks with no change in pain or numbness my doctor was leery about prescribing me Lyrica because the pain wasn't extreme. He said the neuropathy symptoms are likely to disappear within a year of completing my treatment. I'm currently 10 months beyond treatment and no change in numbness.

Avoid the cold/keep warm	Extra warmth use gloves at night to sleep or wear on cool days—Helpful. Heating pad—PRN—Somewhat helpful but I find it short term help.
	Always try to keep my hands and feet warm and rub them with lotion. To help the circulation. Wear socks more often especially when I sleep. Sleep on heating pad to keep warm and relieve joint aches. Drinking warm drinks is comforting. Wear gloves on cold days to keep hands warm.
	My toes are still numb/tingly. Since chemo when my feet get cold they cramp badly. Foot massages give relief while being massaged but comfort goes away when massaging ends. To relieve cramps I cover feet w/ heating pad on high.
	I have numbness in my fingertips only when they get cold. I have muscle weakness in my fingers (doing things like opening lids, containers, writing etc.) when they are cold. The weakness is significant. I have a decrease in fine motor skills when my fingers are cold. At normal temperatures. I have no symptoms. It is a definite change in how they were prior to the chemo. My feet are fine I have no remaining symptoms. To relieve the numbness and weakness I just try to get them warm by covering them or running them in hot water. At work if my hands are cold, I have trouble writing, opening supplies, and so forth. from weakness.
	Cold hurts my fingers and toes so I wear socks and gloves a lot

Keep moving	I walk bike and do deep water aerobics to help I think it helps!!
	Maintained pre-chemotherapy fitness program: 3×/wk jog 1.5–2 miles + lift weights. Maintained pre-chemotherapy activities: swimming and golf weather permitting. For balance practice standing on 1 leg and alternating legs.
	I continue my yoga and triathlon training-swim, bike, run, and weights.
	After taking the supplements and a lot of exercise it slowly went away mostly. It comes back when I get cold.
	Muscle weakness, exercise seems to help
	Walking, water aerobics and free weights were most effective to rebuild strength
	Foot massage, not very helpful. Exercise more helpful than massage.

Massage or rub the affected area	Back rubs, especially with electric massages can be quite efficacious, especially from base of spine and down upper leg.
	Have attempted massage of feet where discomfort is most severe. Gives Temporary relief
	Foot massages give relief while being massaged but comfort goes away when massaging ends.
	If I forget a dose (of Neurontin) I get stabbing pains in my fingers. Hand massages help somewhat.
	I have numbness to feet and achy legs and only feel the pain when I'm sitting or when I go to bed—some days I feel it more than others to relieve the pain I usually rub my feet and if it is persistent I take a pain pill that usually helps me get to sleep.

Live with it	Physical therapy not helpful rubbing massage not helpful heat not helpful Continue to suffer from all of above symptoms haven't been doing anything because it comes and then goes away
	Doctor said there is no cure or relief.
	I have fairly severe numbness in my feet and moderate tingling/numbness in my finger tips. I have not taken any measures to alleviate tis problem, because I haven no idea how to proceed. I have spoken to my oncologist, but he offered no solution.
